# Evaluation of Artificial Intelligence for Patient Self-Triage: Comparison of General-Purpose AI Platforms With the NHS 111 Online Symptom Checker in the United Kingdom

**DOI:** 10.7759/cureus.97834

**Published:** 2025-11-26

**Authors:** Hannah L Brown

**Affiliations:** 1 Independent Research, East and North Hertfordshire NHS Trust, Hertfordshire, GBR

**Keywords:** artificial intelligence, emergency department, nhs 111, self-triage, symptom checker

## Abstract

Emergency departments (EDs) in the UK face substantial pressure due to non-urgent attendances. This technical report evaluates the performance of two general-purpose artificial intelligence (AI) platforms (ChatGPT GPT-5 (OpenAI, San Francisco, California, USA) and Gemini AI v2.5 Flash (Google, Mountain View, California, USA)) for patient self-triage, compared with the NHS 111 online symptom checker. Ten simulated patient scenarios, including five emergency and five non-emergency cases, were assessed against National Institute for Health and Care Excellence (NICE) guideline-based gold standards. Both AI platforms correctly identified all emergency cases; NHS 111 under-triaged one acute emergency. For non-emergency scenarios, AI occasionally over-triaged, recommending emergency assessment for pyelonephritis, whereas NHS 111 correctly classified all non-emergencies. AI triage responses were faster and required fewer follow-up questions than NHS 111, although sometimes producing unclear recommendations. The findings suggest that general-purpose AI may serve as an adjunct to NHS 111, supporting patient self-triage and potentially reducing ED burden. Future work should include larger-scale testing, real patient data, and prospective safety evaluation to assess clinical feasibility.

## Introduction

In recent years, emergency departments (EDs) across the UK have faced increasing strain due to rising attendances, with a substantial proportion of these cases not requiring emergency assessment [1,2]. Machine learning models are widely used in healthcare for tasks such as interpreting medical imaging, identifying patterns, classifying skin lesions, and predicting disease [3,4]. Several studies have explored the potential use of artificial intelligence (AI) in triage assessment, including digital self-triage tools such as eTriage, which have demonstrated variable accuracy, under- and over-triage, and challenges in real-world implementation [5,6]. These studies highlight limitations of existing tools, such as small sample sizes, rule-based decision logic, and a lack of evaluation in general-purpose AI platforms. The only widely accessible self-triage tool in England is the NHS 111 online symptom checker, which uses a rule-based algorithm that directs patients through predetermined pathways, rather than using AI-based prediction of large language models (LLMs) [7]. By contrast, general-purpose AI platforms generate recommendations based on data patterns and may interpret symptoms more flexibly, but their performance for safe patient self-triage remains largely untested. To date, there has been limited evaluation of general-purpose AI platforms in distinguishing emergency from non-emergency presentations, or how they compare with the established NHS 111 online symptom checker. Addressing this gap could support patients in safely assessing their symptoms at home, potentially reducing unnecessary ED attendances and improving resource allocation [5].

This report evaluates the performance of two AI platforms, ChatGPT GPT-5 (OpenAI, San Francisco, California, USA) and Gemini AI v2.5 Flash (Google, Mountain View, California, USA), in comparison with the NHS 111 online symptom checker, using 10 simulated patient scenarios to assess their ability to distinguish emergency from non-emergency presentations and provide safe self-triage recommendations.

## Technical report

Study design

A vignette-based simulation study was conducted to compare the triage recommendations of AI platforms and the NHS 111 symptom checker against UK clinical guidance. A sample of 10 vignettes was chosen for feasibility as a proof-of-concept evaluation. Ten patient vignettes were developed by the author, a UK-trained physician, based on common clinical presentations (see Appendix), including age, gender, and a brief symptom description representative of typical user input. The vignettes were based on specific clinical cases spanning several specialties, including ophthalmology, obstetrics/gynaecology, dermatology, orthopaedics, general medicine, and paediatrics; children under five were excluded, as the NHS 111 symptom checker does not cover this age group.

Vignette Classification

Five vignettes represented non-urgent diagnoses suitable for self-care or community management. These vignettes were written to exclude guideline-defined red-flag features that would require higher-acuity referral. For example, the gastroenteritis case described self-limiting symptoms without dehydration, haematemesis, abdominal rigidity, or other features suggestive of acute abdomen, ensuring clear delineation between non-urgent and emergency presentations for the purposes of controlled comparison. Five vignettes represented acute urgent presentations requiring ED assessment.

The gold standard for determining whether each vignette required acute (ED) or non-acute care (Table 1) was based on the relevant sections of NICE guidelines [8]. Each vignette was independently reviewed by the author, a UK-trained physician with emergency medicine experience, and classified as ED or not ED. Classifications were based primarily on the relevant NICE guidelines [8], considering presenting symptoms, risk factors, and guideline-specified red flags, with the physician’s review ensuring appropriate application of the guidelines to each case. If multiple management options were plausible, the highest-acuity recommendation consistent with the guidelines was recorded.

**Table 1:** Diagnoses for vignettes with gold standard classification and justification

Vignette diagnosis	Gold standard	Justification
Pulmonary embolism	Emergency department	Dyspnoea, chest pain, symptoms suggestive of deep vein thrombosis, and risk factors of active cancer, arrange emergency admission.
Acute angle closure glaucoma	Emergency department	Acute eye pain, red eye with impaired visual acuity, to be admitted immediately for assessment.
Ectopic pregnancy	Emergency department	Abdominal pain with missed period and vaginal bleeding. Red flags, risk of life-threatening ectopic pregnancy, immediate ED assessment
Stroke/TIA	Emergency department	Unilateral weakness or dysphagia, requires immediate emergency admission for suspected stroke.
Sepsis	Emergency department	Change in cognitive function, fever, and risk factor for sepsis (>75, frail), requires immediate hospital admission.
Skin rash – food allergy	Not emergency department	No red flags for anaphylaxis, rash from suspected food allergy can be assessed routinely in the community.
Mild gastroenteritis	Not emergency department	Diarrhea, nausea, and abdominal pain, risk factors of recent takeaway food. No features of severe dehydration and taking oral fluids, therefore, can be managed in primary care.
Ankle sprain	Not emergency department	Able to bear weight and no red flags that would require referral to the emergency department. To be advised on self-management strategies in the first instance.
Pyelonephritis	Not emergency department	Flank pain, fever, and urinary symptoms suggestive of pyelonephritis. No signs of sepsis or guideline-specified red flags for admission were present. The case was classified as suitable for outpatient assessment and antibiotic management in general practice, assuming timely access to care, as delays could increase the risk of progression to sepsis.
Viral upper respiratory tract infection (URTI)	Not emergency department	Sore throat and runny nose, no features of a serious complication. Can be managed with self-care.

AI Platform Testing

Two large language models were evaluated: OpenAI ChatGPT (GPT-5) and Google Gemini AI (v2.5 Flash). Platforms were accessed via publicly available web interfaces (ChatGPT: chat.openai.com; Gemini AI: gemini.google.com) with default settings; memory and plug-ins were disabled. No custom instructions or temperature adjustments were used. Each vignette was entered in a new temporary chat with no additional context. Responses to follow-up questions were restricted to information provided in the vignette; unreported symptoms were assumed absent. For one vignette in a single AI platform, a uniform "yes" was provided to a follow-up question solely to allow the model to continue its response and generate an outcome. This did not add clinical information or influence the triage classification. All AI conversations were documented verbatim; once an action was advised, the conversation ended.

NHS 111 Symptom Checker Testing

NHS 111 uses a rule-based algorithm based on NICE guidance. Vignettes were used to answer all questions as accurately as possible. Predetermined answer options “unknown”/“I’m not sure” were used for unaccounted information. A fixed postcode (SW1A 2AA, Downing St.) ensured consistent service availability.

Outcome Classification

Outputs were classified as ED or not ED. Lower-acuity options included self-care at home, same-day GP appointments, or urgent treatment centre visits. If multiple actions were suggested, the highest-acuity option was used as the safest interpretation.

Each output was labelled: 1) matched: agreed with the gold standard; 2) over-triaged: higher-acuity recommendation than the gold standard; 3) under-triaged: lower-acuity recommendation than the gold standard.

Ethics

No human participants or identifiable patient data were used; all cases were simulated. Formal institutional review board (IRB) or ethics approval was not required.

Testing Dates

All AI and NHS 111 testing was conducted between September 20 and 25, 2025.

Results

NHS 111 Symptom Checker Questions

Three emergency cases (PE, ectopic pregnancy, stroke/TIA) were flagged immediately on the opening page, while other vignettes required eight to 22 questions to reach a decision (Table 2).

**Table 2:** Number of questions asked for each vignette by the NHS 111 symptom checker

Vignette	No. of questions asked by the NHS 111 symptom checker
Pulmonary embolism	None – flagged on the first page. Call 999 for chest pain.
Acute angle closure glaucoma	8
Ectopic pregnancy	8
Stroke/transient ischemic attack (TIA)	None – flagged on the first page. Call 999 signs of a stroke.
Sepsis	None – flagged on the first page. Call 999 for sudden confusion.
Skin rash – food allergy	22
Mild gastroenteritis	20
Ankle sprain	9
Pyelonephritis	13
Upper respiratory tract infection (URTI)	13

AI Platforms

ChatGPT asked a single follow-up question for one vignette (ankle sprain). Gemini AI generated responses without follow-up questions. All AI responses were generated using only the information provided in the vignette.

Overall Accuracy Across Platforms

The total outputs were 30 (10 vignettes × 3 platforms): 1) 27 matched the gold standard, 2) one was under-triaged, and 3) two were over-triaged.

For under-triaged outputs, acute angle closure glaucoma by NHS 111 (recommended NHS 111 nurse call instead of immediate ED attendance). For over-triaged outputs, pyelonephritis was flagged as requiring ED by ChatGPT and Gemini AI.

Gemini AI suggested lower-acuity options for pyelonephritis and ectopic pregnancy; these are summarized in Table 3. 

**Table 3:** Performance summary and lower-acuity options

Platform	Matched gold standard	Over-triaged	Under-triaged	Notes on lower-acuity options
NHS 111 symptom checker	9/10	0	1	None
ChatGPT	9/10	1	0	None
Gemini AI	9/10	1	0	Suggested lower-acuity options in two instances

## Discussion

Accuracy and triage performance

Overall, 90% of the outputs matched the gold standard classification. All AI platforms correctly identified the majority of emergency cases (Table 4). The NHS 111 symptom checker under-triaged one emergency vignette (acute angle closure glaucoma), recommending a call with an NHS 111 nurse rather than immediate ED attendance. While this deferred decision provides a safety net, it highlights that rule-based symptom checkers may not autonomously identify all emergencies.

**Table 4:** Gold standard “emergency department” cases and triage outcomes across platforms TIA: transient ischemic attack, ED: emergency department

Vignette diagnosis	NHS 111 symptom checker	ChatGPT	Gemini AI
Pulmonary embolism	ED	ED	ED
Acute angle closure glaucoma	Not ED	ED	ED
Ectopic pregnancy	ED	ED	ED
Stroke/TIA	ED	ED	ED
Sepsis	ED	ED	ED

For non-emergency vignettes (Table 5), NHS 111 correctly classified all cases. Both AI models over-triaged the pyelonephritis vignette, consistent with previous research indicating that general-purpose language models tend to adopt a cautious approach compared to dedicated symptom checkers [9]. ChatGPT referenced potential warning signs of a serious infection, while Gemini AI explicitly noted possible progression to kidney damage or sepsis. These findings illustrate that AI recommendations often reflect potential risk rather than current presentation, which must be considered when using AI for triage. Determining appropriate risk thresholds is challenging; overly cautious triage increases unnecessary ED attendances, while insufficient caution risks delayed recognition of serious conditions [10]. Using a guideline-based gold standard ensures objective evaluation of AI performance against clinically accepted criteria.

**Table 5:** Gold standard "not emergency department" cases and triage outcomes across platforms URTI: upper respiratory tract infection, ED: emergency department

Vignette diagnosis	NHS 111 symptom checker	ChatGPT	Gemini AI
Skin rash - food allergy	Not ED	Not ED	Not ED
Mild gastroenteritis	Not ED	Not ED	Not ED
Ankle sprain	Not ED	Not ED	Not ED
Pyelonephritis	Not ED	ED	ED
Viral URTI	Not ED	Not ED	Not ED

For example, a UK study of the digital self-triage system eTriage found only a weighted kappa of 0.14 (95% CI 0.14-0.15) when compared with nurse triage, with under-triage at 10.1% and over-triage at 59.2%. While this study evaluated a digital self-triage tool rather than a general-purpose AI system, this comparison highlights that even non-AI digital triage tools face substantial accuracy challenges, underscoring the need for rigorous benchmarking and evaluation of AI systems [6].

LLM-based systems are also prone to bias, hallucination (the generation of plausible but incorrect information), and prompt sensitivity, all of which can affect triage reliability. These limitations highlight the need for rigorous validation, transparency, and human oversight before any clinical integration.

Lower-acuity recommendations and patient safety

Gemini AI suggested multiple recommendations in two cases, including lower-acuity options such as same-day GP appointments or urgent treatment centre visits (Table 3). Multiple options may create ambiguity for users, who could choose the lowest-acuity recommendation inappropriately, potentially delaying care. Both over-triage and under-triage present patient safety risks: under-triage may delay assessment in acute presentations, increasing morbidity and mortality, while over-triage can divert non-urgent cases to the ED, straining resources. Effective digital triage should provide a single, clear, and actionable recommendation to optimize patient safety and resource allocation.

Limitations and strengths

This pilot study used a small sample of ten simulated vignettes and three platforms, which limits generalisability across the full range of clinical presentations. This sample size provided controlled proof-of-concept testing across a mix of presentations. Larger studies incorporating additional vignettes and real-world patient inputs are needed to confirm these findings. All vignettes were reviewed by a single author, which may introduce bias, although classifications were based strictly on NICE guidelines to minimise subjectivity.

The gold standard classifications were based on NICE guidelines rather than real patient outcomes, so real-world over- or under-triage performances remain unknown.

Although written in lay language, the vignettes may not fully reflect how real patients describe symptoms. Temporary chats were used to prevent memory effects, though conversational memory in practice could alter responses. Each vignette was tested once per AI platform; repeated testing was not performed, so outputs may vary between runs. Future work could include repeated AI queries to assess consistency and variability in recommendations. AI models are rapidly updated, so future versions may perform differently. Laboratory or imaging data were not included, reflecting real-world conditions where patients performing self-assessment typically lack this information.

The study evaluated multiple platforms across a variety of clinical specialties, with a gold standard based on the NICE guidelines [10]. The vignette-based methodology allows for reproducibility and scaling with more cases or platforms. This design provides an objective comparison of AI and rule-based symptom checkers in a controlled setting.

Implications and recommendations

General-purpose AI platforms show potential as an adjunct to NHS 111 for patient self-triage, but current models are not sufficiently reliable for standalone use. Over- and under-triaging highlight the importance of clear guidance and user instructions. AI-based triage tools have potential clinical and medico-legal implications. Incorrect recommendations-whether over- or under-triage-may delay care or lead to unnecessary service use. As responsibility for clinical decisions currently rests with human practitioners, AI systems should be viewed strictly as decision-support tools rather than autonomous triage mechanisms. Ensuring human oversight remains essential to patient safety. Future work should include larger datasets, real patient inputs, prospective safety evaluation, and assessment of conversational memory impacts to determine clinical feasibility.

## Conclusions

EDs in the UK remain under substantial pressure, with a significant proportion of non-urgent attendances contributing to demand. This pilot study evaluated 10 vignettes across two general-purpose AI platforms (ChatGPT and Gemini AI) and the NHS 111 online symptom checker to explore their potential ability to support patient self-triage at home. AI demonstrated correct identification of all five emergency cases, but struggled with some non-emergency presentations, occasionally recommending higher-acuity assessment than necessary (over-triage) or suggesting multiple conflicting actions. The NHS 111 symptom checker performed consistently, correctly identifying nine out of 10 cases, with under-triage occurring in one emergency case (acute angle closure glaucoma). These findings suggest that AI may have potential as a supportive triage tool but must be refined and validated before integration into real-world pathways.

The strengths of this study include the direct comparison of general-purpose AI platforms with the NHS 111 symptom checker in a UK-relevant context, which has not been previously reported. Limitations include the small number of vignettes and the use of simulated patient inputs rather than real patient queries. AI offers efficiency by producing rapid outcomes with minimal questioning, whereas the NHS 111 symptom checker adds value through a structured process designed to identify red flags. Future approaches could consider combining the strengths of both systems. If developed and validated, AI could complement existing triage systems, potentially reducing unnecessary ED attendances, improving resource allocation, and supporting patients to make safer decisions about care. While AI tools may offer educational or decision-support value, their current performance indicates they should complement, not replace, clinician-led triage.

Future research should involve larger, prospective studies using real patient queries, with defined endpoints such as triage accuracy, rates of over- and under-triage, and patient-safety outcomes. Evaluation of usability, user experience, and patient comprehension will also be important, alongside assessment of clinical validity and alignment with regulatory standards. Ultimately, AI triage tools are best viewed as decision-support systems that may complement existing services by supporting patient self-management and reducing demand, but current evidence remains preliminary.

## Appendices

Patient vignettes developed by the author

Vignette 1: Pulmonary Embolism

I am a 50-year-old female with breathlessness and chest pain since this morning. My calf has also been hurting for a couple of days. I have metastatic breast cancer. What should I do?

Vignette 2: Acute Angle Closure Glaucoma

I am a 60-year-old man with sudden eye pain, it's red, and my vision is blurred. I am also diabetic. What should I do?

Vignette 3: Ectopic Pregnancy

I am a 23-year-old female with lower abdominal pain and some vaginal bleeding. My period was due six weeks ago. I am normally well. What should I do?

Vignette 4: Stroke/TIA

My mum is a 70-year-old lady and suddenly felt unwell; her face has drooped on one side, and she is not talking to us. She smokes and has high blood pressure. What should I do?

Vignette 5: Sepsis

My dad is an 85-year-old man; he has been unwell for the past week, but overnight, he has become more confused and drowsy. He is very hot to touch. He also has dementia. What should I do?

Vignette 6: Skin Rash - Food Allergy

My son is 6 years old; he has a red rash on his tummy that started after eating lunch yesterday. He is otherwise well and behaving as normal. What should I do?

Vignette 7: Mild Gastroenteritis

I am a 32-year-old man. I have had diarrhoea for the past 24 hours following a takeaway meal, with a tummy ache and feel a bit sick. I don’t feel like eating, but am managing to drink. I am normally well. What should I do?

Vignette 8: Ankle Sprain

I am a 15-year-old male and fell while playing football earlier today. My ankle hurts and is a bit swollen, but I can stand on it. What should I do?

Vignette 9: Pyelonephritis

I am a 35-year-old female. I have had pain when going for a wee for the past two days, and now in my back. I feel shivery and have a fever of 38 degrees Celsius. What should I do?

Vignette 10: Viral URTI

I am a 26-year-old male with a scratchy throat and cough for the past four days. I have a runny nose, but am otherwise well. What should I do?
